# How COVID-19 has Been Transforming the Notion of Care

**DOI:** 10.17533/udea.iee.v38n2e01

**Published:** 2020-07-10

**Authors:** María Victoria López López

**Affiliations:** 1 Sociologist, Masters. Professor, Social Policies and Health Services Research Group, Faculty of Nursing at Universidad de Antioquia, Medellín, Colombia. Email: maria.lopez@udea.edu.co Universidad de Antioquia Universidad de Antioquia Medellín Colombia maria.lopez@udea.edu.co

The year 2020 surprised us with COVID-19, which expanded throughout Asia and Europe and became a pandemic, reaching Latin America and our cities and everything seemed to change, including the most intimate and private relationships of social life in general, with repercussions in the human condition that lead to thinking about its setbacks. For the population not expert in epidemiology, it results quite difficult to understand what is happening and know what to do and how to assimilate discourses that break into the private sphere and - at the same time - amalgamate with social, environmental, economic, political, and other problems that even lead epidemiologists themselves to new reflections they thought already overcome. But also, within this context positive issues are unveiled, like solidarity, reflecting on consumerism, and caring for nature.

We are told: life is most important and that is how we have assumed it from the confinement, although sometimes we are assailed by the question of whether we are reducing life to a survival detached from human dignity, wellbeing, safety, peacefulness, feelings, and emotions. The only thing that is undeniable is recognizing that today we live an unthinkable reality, which threatens with the loss of human beings under conditions of an explicit incapacity of health services to provide the necessary care; ecological, economic deterioration is exacerbated, affecting relationships and exchange among countries and among people and revealing, not because of this virus, but as effects of a crisis of the economic-political system in its distinct dimensions, the rawness of profound social debts expressed as inequities, unemployment, hunger, corruption, and intra-family violence among many other problematics.

Within this framework, theories on health and disease, as biological-psychological-social process, become increasingly more necessary to bring us closer to understanding the effects of this pandemic beyond statistical and numerical readings, such as the need to flatten “the epidemiological curve”, given that although time is gained to equip the health services and to make political and health decisions, the virus continues and its effects will possibly continue for much more time with consequences not only on health, as morbidity/mortality-centric fact, but as human and social relations constructed on a long-lasting process.

Probably, this health crisis indicates the need to delve further into understanding the relationship among the biological, individual, social, and the health policy, as well as to delve into the analysis of the same health-disease concept as human vital process. Likewise, with the pandemic, there is no mechanical opportunity in sight to make political, economic, social, or personal changes. That is only possible if transformations are adopted in the micro and in the macro, in the general and in the particular, in the individual and the social. This is the framework in which the present reflection is inscribed, which - within the scenario of academic life - inquires on the changes suggested by the current situation on caring: object of the nursing profession.

Currently, health takes on an unusual sense, it is the subject of debates in different spheres despite the setbacks that exist in a privatized health system like ours and regulated by market laws. Day by day, care is a slogan proclaimed by civil and military authorities, the media, and civil society, pointing out that we all have to take responsibility to avoid contagion, through hand washing and social isolation and in regard to this general matter, the responsibility for the maintenance of life is placed in upon everyone; this task is the most decisive action of daily life today, it is a type of self-care to care for oneself and care for others (in our case, fundamentally those over 70 years of age).

Within this framework, health care becomes a fundamental issue and I consider that nursing has much to contribute to highlight it as a matter of life involved with the rights of people, with the cross-section between scientific knowledge, culture and the subjectivity of patients, families, and communities. As an object of training and practice of nursing, care does not concentrate merely on making and displaying knowledge or technical skills, but rather establishing connections, bonds, a dialogical, reciprocal condition, committed to caring for others and offering alternatives for physical well-being, autonomy, self-knowledge, and the capacity for self-care.

More than an isolated action, nursing care is a living act, an essential process in the profession, given that, as stated by authors, like Salazar,(1) Malváez and Castrillón,(2) it requires not only human-human transactions, but also their own knowledge, dedication, values, and the recognition that it is a personal, ethical, and moral relationship between the person receiving care and who offers it. As proposed by Collière,(3) based on respect for people and humanity, care is concretized in the mobilization of vital resources to help live, promote life and this definition reaffirms the value of professional care that as inter-relational activity seeks to recover and maintain closeness with sick and healthy people, with families and communities through direct and comprehensive care.

In no way does what happens today in society with care assigned to individual and family responsibility within the homes replace or minimize professional nursing care; on the contrary, it revalues it and indicates that during and after this pandemic these professionals will play a fundamental role to make care more meaningful and, thereby, the need to enhance the relationship with citizens perhaps more disciplined and trained in asepsis, but more in need of comprehensive care that takes into account the biological, psychological, and social in which emerge learning and adverse effects of a social change as abrupt as the one we are experiencing.

Loving and rigorous and politically fair and inclusive health care is precisely what is revealed as lacking under a social system anchored in market values. This is a call to nursing professionals and students to envision future particularities of health care that in some cases is postponed to other times because the central demands of the current situation of COVID-19 so require it. Thus, is the case of caring for patients with chronic diseases, care responses to the deterioration of mental health among other situations. Similarly, it is fitting to recover creative and positive actions that, amid the contingency, populations have built what calls for strengthening a dialogue of knowledge and practices of popular and professional care. In the same manner, it is also necessary to reflect about the limitations this crisis supposes on health promotion actions and on life within the framework of comprehensive care.

Another key problem nursing researchers, such as De la Cuesta(4) and Aiken *et al.,*(5) had highlighted during non-pandemic times, which is currently gaining strength in conditions of social isolation, is the challenge of providing professional nursing care closely related to the informal care carried out at home or in some institutions for the protection of the elderly. For these researchers, it is worrying to observe that a large part of health care occurs in the private sphere and that the family is increasingly protagonist as a caregiver at home and on occasions more and more frequent in hospital institutions; naturally, it is assumed by nursing professionals and health institutions that nursing care be frequently left in the hands of untrained family caregivers. Said situation impacts upon patients’ companions who assume as workers an important part of the care under conditions of loneliness, lack of knowledge, technical and human difficulties, as well as fear of being wrong or concern about not knowing how to do what is correct, which translates into conditions of risk for the safety of patients and their companions.

Concern for the participation of the patient’s family or companions during the care process when they lack resources, knowledge, and skills, as strategy to complement professional resources in health services, is taken as an indicator of poor quality in nursing care and as a risk to patients. This situation sometimes responds the need to make up for staff cuts in hospital institutions and translates into work overload for nursing professionals.

Amid the uncertainty and chaos of the pandemic, the current condition suggests in the health field and specifically in nursing to meet the broad notions of care proposed by authors, like Boff(6) who proposes that "Caring is more than an act, it is an attitude; therefore, it covers more than a moment of attention, of zeal and concern, it represents an attitude of occupation, responsibility and affective commitment with the other. Attitude is a source, which generates many acts and expresses the underlying attitude”.

In fruitful dialogue among different types and sources of care for life, professional care has an opportunity and a responsibility to contribute to confronting a complex and contradictory context of great possibilities for well-being, coexistence, and of limited scope, which radiate to all in conditions of equity, rights, capacities, and potentialities. (7) In this sense, the proposal is an approach to the category of care based on empathy and compassion, from a broad field that permits identifying particularities and connection points with nursing care defined as object of the profession.

According to Carrasco, Borderías and Torns,(8) the concern for understanding care in societies is a task that increasingly gains more importance and which requires interdisciplinary approaches. In the health field, there has always been a multiplicity of care, in response to the way of understanding the causes and effects of problems that affect the body, the psyche, or the development considered "normal" in groups or individuals. These cares include popular knowledge, family care, and other social practices aimed at maintaining or restoring the health of people and of the planet. Likewise, nursing care, which also had its origin in the domestic sphere, under the responsibility of women and which, only from modern nursing, is considered the profession *par excellence* aimed at offering health care to people, families, and groups.

Although the daily, popular and informal care, and nursing professionals are supported by sources of different origin, they are fields that - in practice - overlap and sometimes bear tension or complementary relationships. As tension, on some occasions, popular care is unknown or confronted from the professional side and, when recognized as a complement, they are alternatives of dialogical, interdisciplinary practices and a possibility to broaden the field of the nursing profession and enhance their practice as educators beyond their own students, toward informal caregivers and those being cared for. Thus, it is expected that the current situation on which this reflection is centered, permits nursing professionals to continue with theoretical and practical elaborations about the humanization of care, which evidence the positive aspects reached in the relationship of nursing care and care at home that this contingency is assigning as individual, family, and social responsibilities.

Likewise, it is an invitation to those in the formation process in this discipline to reflect on the challenges the social and health reality supposes on the professional formation with new experiences and new questions. It is necessary to document at this time the particularities of health care in the institutional and in the community, barriers to communication and interaction in some scenarios, like the rural, and even in the academic among professors and between these and students. Likewise, it is necessary to generate proposals that from the global and local (*glocal*) seek international health agreements that overcome market barriers and privilege the human condition.

Finally, the need is identified for professionals and students to recognize each other as actors in caring, public health, of collectives, and in education for health and to reflect on caring for nurses, a matter that is materialized under working conditions, biosafety, recognition, protection, and guarantee of rights as citizens and as health workers.
